# Supplementary material for: The causal relationship between the human gut microbiota and pyogenic arthritis: a Mendelian randomization study
Source: Front Cell Infect Microbiol. 2024 Nov 26;14:1452480. doi: 10.3389/fcimb.2024.1452480 (PMC11629706; doi:10.3389/fcimb.2024.1452480)
Supplement: Supplementary file 1 [file DataSheet1.zip › mendelian test/ebi-a-GCST90027842.csv_forest.pdf]

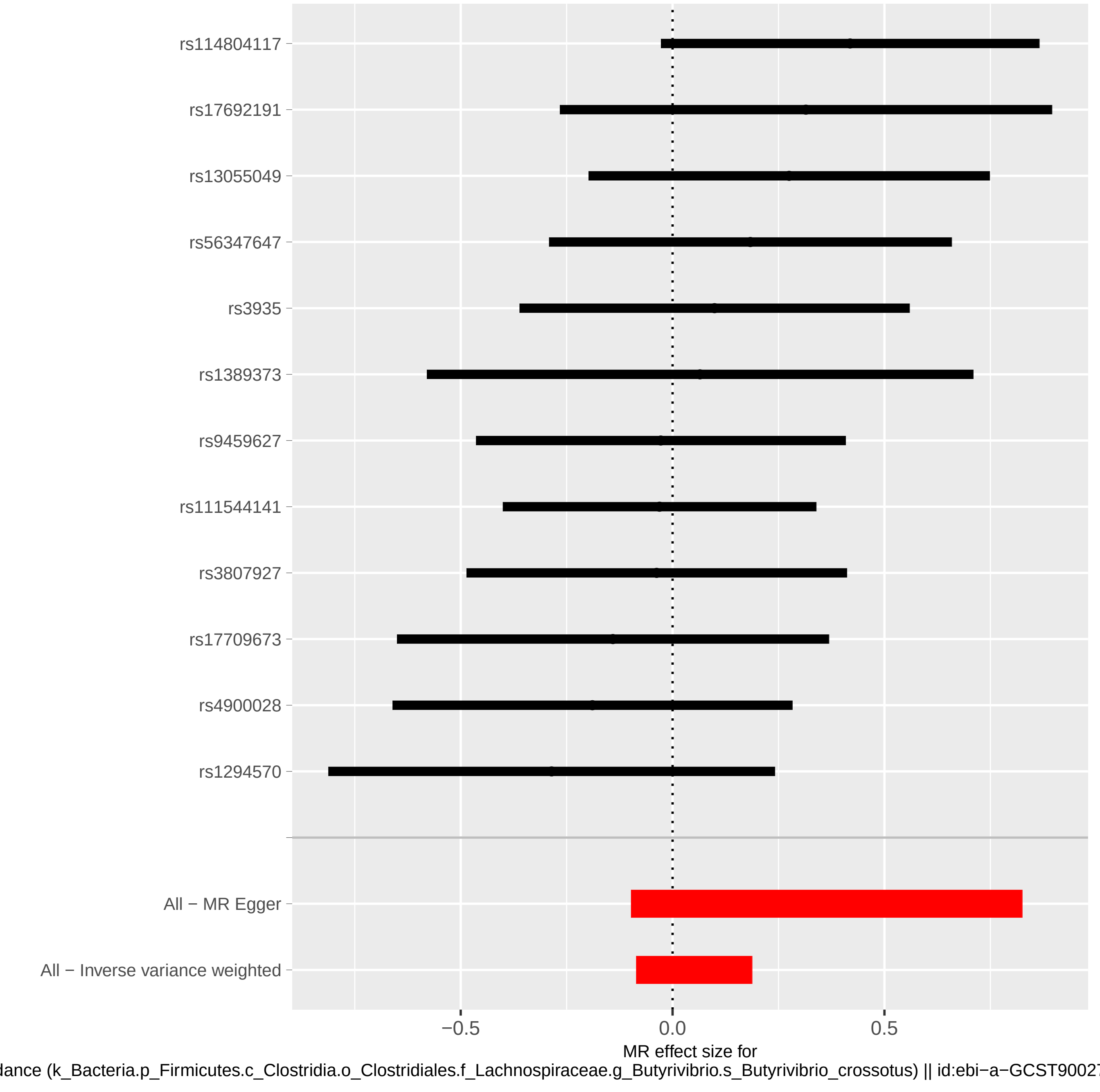

rs114804117
rs17692191
rs13055049
rs56347647
rs3935
rs1389373
rs9459627
rs111544141
rs3807927
rs17709673
rs4900028
rs1294570
All − MR Egger
All − Inverse variance weighted
−0.5
0.0
0.5
MR effect size for
dance (k_Bacteria.p_Firmicutes.c_Clostridia.o_Clostridiales.f_Lachnospiraceae.g_Butyrivibrio.s_Butyrivibrio_crossotus) || id:ebi−a−GCST9002
